# Increased aortic intima-media thickness may be used to detect macrovascular complications in adult type II diabetes mellitus patients

**DOI:** 10.1186/s12947-018-0127-x

**Published:** 2018-06-12

**Authors:** Ayse Selcan Koc, Hilmi Erdem Sumbul

**Affiliations:** 1 Department of Radiology, University of Health Sciences - Adana Health Practices and Research Center, Adana, Turkey; 2 Department of Internal Medicine, University of Health Sciences - Adana Health Practices and Research Center, Adana, Turkey

**Keywords:** Aortic intima-media thickness, Macrovascular complication, Type II diabetes mellitus

## Abstract

**Background:**

Carotid intima media thickness (C-IMT) and aortic IMT (A-IMT) increase in adult and pediatric patients with diabetes mellitus (DM), respectively. In both age groups IMT is used for early detection of macrovascular complications. In adult DM patients, A-IMT is still not a routine examination and is not used frequently. We aimed to determine whether there is an increase in A-IMT values measured from abdominal aorta besides traditional C-IMT in patients with type II DM and to determine parameters closely related to A-IMT in the same patient group.

**Methods:**

We included 114 type II DM patients and 100 healthy control subjects similar in age and sex in our study. Bilateral C-IMT and A-IMT values were measured by B-mode ultrasonography (USG) in addition to anamnesis, physical examination and routine examinations of all patients.

**Results:**

When the clinical, demographic and laboratory data of patients with and without DM were compared, there was a high level of glucose and HbA1c and low hemoglobin levels in the DM patient group. All other parameters were found to be similar between the two groups. When the B-mode USG findings were examined, it was found that C-IMT and A-IMT were increased in patients with DM, with the A-IMT increase being more prominent. A-IMT values were found to be strongly and positively correlated with age, systolic blood pressure, blood urea nitrogen, DM onset time and HbA1c levels, and a negatively and significantly correlated with hemoglobin levels (*p* < 0.05, for each). In the regression model, the parameters correlating most closely with A-IMT were DM diagnosis onset time, HbA1c and hemoglobin levels (*p* = 0.001 and β = 0.353, *p* = 0.014 and β = 0.247 and *p* < 0.001 and β = − 0.406).

**Conclusions:**

As in pediatric DM patients also in adult DM patients A-IMT can easily be measured with new model USG devices. A-IMT must be measured during abdominal USG which is routine in adult DM patients. A-IMT is an easy, reproducible and non-invasive parameter that may be used in the diagnosis of macrovascular complications of adult type II DM.

## Background

Type II diabetes mellitus (DM) is a common metabolic disease, causing macrovascular and microvascular complications. DM is a major and known risk factor for atherosclerosis development. The most important macrovascular involvement in DM is coronary artery disease (CAD). Increased carotid intima-media thickness (C-IMT) in DM patients is closely related to asymptomatic or subclinical atherosclerosis and is recommended as a routine examination [1, 2].

The artery wall contains three layers; tunica intima, tunica media and tunica adventitia. The atherosclerotic process occurs in the first two walls, resulting in a structural change in the early period as an increase in IMT thickness. IMT on the posterior wall is clearly distinguishable with ultrasonography (USG). C-IMT and aortic IMT (A-IMT) increase in adult and pediatric patients with DM, respectively [1, 2]. IMT is used in early detection of macrovascular complications in both groups. Studies about the A-IMT evaluation obtained from abdominal aorta in adult DM patients are limited in the literature [3, 4]. For this reason, the importance of A-IMT in adult DM patients is unknown, it is not a routine examination and is not used.

A-IMT measurement can be used to detect the development of early atherosclerosis, since atherosclerosis is first started in the distal abdominal aorta [5–7]. Therefore, we hypothesize that A-IMT obtained with new model high-resolution USG devices may be more useful for early detection of macrovascular complications in adult DM patients than C-IMT. In our study, we aimed to determine whether there was an increase in A-IMT values measured from the abdominal aorta in addition to C-IMT, which became a routine for type II DM patients, and to identify parameters closely related to A-IMT in the same patient group.

## Methods

### Study population

We included 114 type II DM patients (mean age: 46.3 ± 12.8 years, male / female: 52/62) and 100 healthy control subjects similar in age and sex (mean age: 47.1 ± 12.3 years, male / female: 48/52) in our study. When we looked in terms of the control group, subjects that have major risk factors that may lead to an increase in IMT as smoking habits, hypertension (HT), hyperlipidemia and obesity were excluded from study*.* Those with secondary or malignant HT, calcific plaques, abdominal aneurysm or dissection, congestive heart failure, cerebrovascular disease, severe heart valve disease, inflammatory diseases, hematologic diseases, cancer, pregnancy and renal failure were also excluded from both groups. The Local Ethics Committee approved the study protocol and each participant gave written informed consent.

After a detailed medical history and a complete physical examination, basic characteristics of patients such as age, gender, HT, current smoking status, family history, and hyperlipidemia, presence of CAD and body mass index (BMI) were recorded.

Plasma glucose, HbA1c, triglyceride, low-density lipoprotein, high-density lipoprotein, hs-CRP, uric acid, creatinine, hemoglobin and white blood cell concentrations were measured using an automated chemistry analyzer using commercial kits.

### Main carotid and abdominal aortic B-mode ultrasonography evaluation

**Fig. 1:**
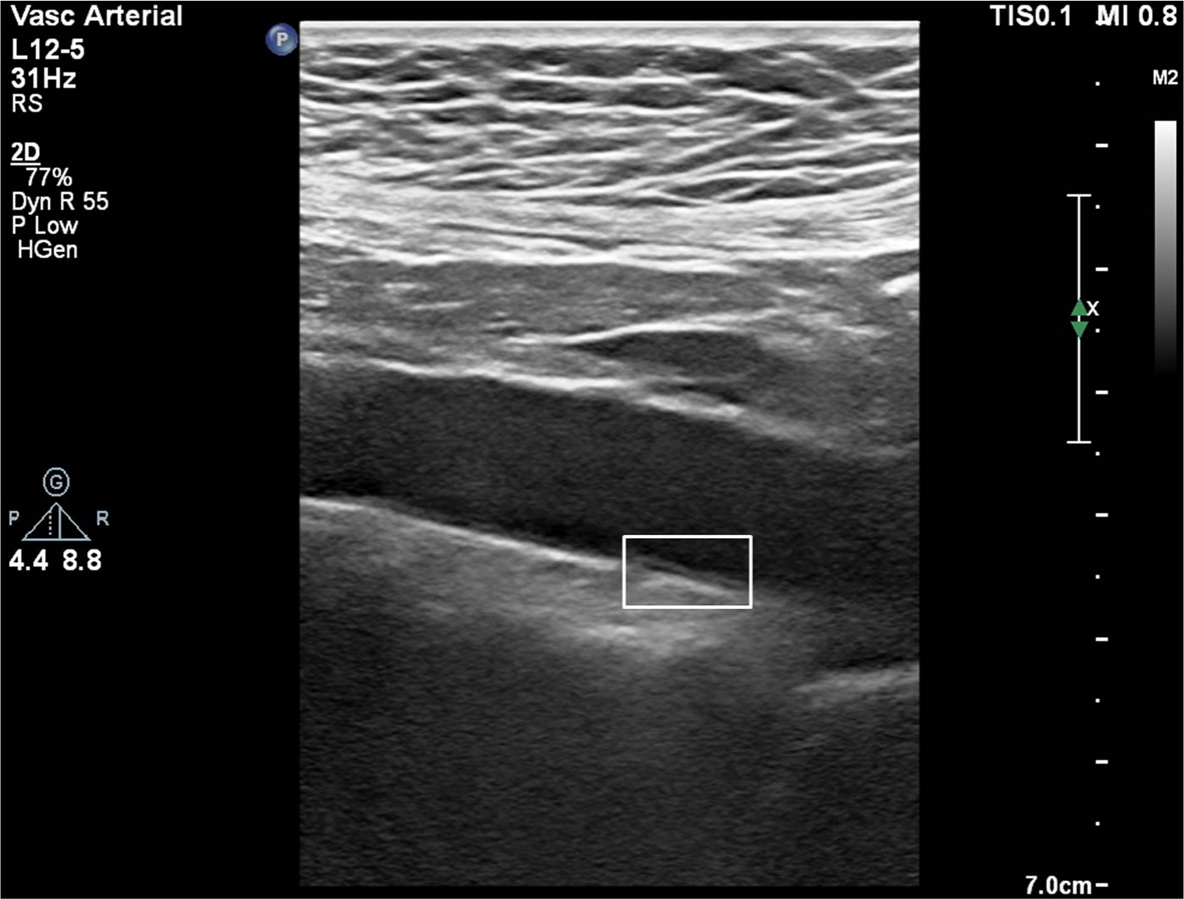
Aortic IMT measurements with B-mode ultrasound from the level of renal artery bifurcation to iliac artery bifurcation

The left and right main carotid artery and abdominal aorta were examined with a high resolution Doppler ultrasound system (Philips EPIQ 7) equipped with a 12 and 5 MHz high resolution linear and convex converter (Philips Health Care, Bothell, WA, USA) respectively. Ultrasound scanner setting was made to be useful for every patient for all B-mode USG examination (gain [55–75 dB]; penetration depth [2.5–16 cm]; dynamics range [50–60] and zoom range [0.8–2.0]). Arteries were examined both longitudinally and transversely. All arteries were scanned longitudinally to visualize the IMT on the posterior or distal artery wall. All measurements were made on frozen images. Two frozen images that the highest quality for the operator opinion’s were selected for analysis in each study. The IMT is defined as the distance from the anterior edge of the first echogenic line to the anterior edge of the second echogenic line. The first line represents the intima-lumen interface and the second line represents the collagen-containing top layer of adventitia. Vascular IMT was measured using ultrasonic calipers in the presence of two independent and blind observers. All IMT values were calculated as averages of six measurements. Patients were examined in supine position. While studying carotid artery patients rotated their heads by *45 °*counter from scanned area*.* IMT, which was measured from the distal wall of the right and left main carotid artery in the 10–20 mm proximal segment before bifurcation, was accepted as C-IMT. Abdominal A-IMT was examined in the 10–15 mm segment through from the level of renal artery bifurcation to iliac artery bifurcation. IMT measured from the posterior wall of the abdominal artery was accepted as A-IMT (Fig. 1). Increased A-IMT and C-IMT accepted as > 2.90 mm and > 0.90 mm respectively [8–10].

### Statistical analysis

All analyzes were performed using SPSS 20.0 (SPSS for Windows 20.0, Chicago, IL, USA). Data are expressed as mean ± SD for continuous variables and as percent for categorical variables. Continuous variables with normal distribution were compared with Student t test, while Mann-Whitney U test was used for not normally distributed samples. Categorical variables and frequencies were compared with chi-square (χ2) test. Statistical significance was defined as *p* < 0.05 for all comparisons. Pearson and Spearman correlations were used to examine the relationship between continuous variables. A multivariate, step-by-step forward conditional linear regression analysis was used to determine parameters much closer to the A-IMT. In the correlation analysis, the important parameters related to A-IMT were selected in the multivariate model. A receiver operator characteristic (ROC) curve analysis was carried out to identify the optimal cut-off point of A-IMT and C-IMT to detect presence of DM.

## Results

### Baseline characteristics

When the clinical, demographic and laboratory data of patients with and without DM were compared, there was a high level of glucose and HbA1c and a low hemoglobin level in the DM patient group. All other parameters were found to be similar between the two groups (Table 1). The incidence of smoking, HT and hypercholesterolemia in DM patients was 41.2, 57.9 and 51.8%, respectively. Patients were followed up for DM for an average of 4.8 ± 2.5 years, and the frequency of oral anti-diabetic and insulin treatment was 70.2 and 39.5%, respectively.

**Table 1:** Baseline characteristics and laboratory parameters in patients with type II DM and controls

	Type II DM *n* = 114	Controls *n* = 100	*p*
Age (year)	46.3 ± 12.8	47.1 ± 12.3	0.666
Gender (male)	62	58	0.435
Heart rate (beat/min)	84.9 ± 15.8	82.8 ± 16.1	0.331
Office systolic BP (mmHg)	132.9 ± 17.7	128.3 ± 8.1	0.069
Office diastolic BP (mmHg)	77.9 ± 8.3	75.9 ± 7.7	0.085
Body mass index (kg/m^2^)	23.5 ± 4.1	23.2 ± 3.7	0.755
White blood cell (μL)	10.1 ± 3.4	9.8 ± 3.8	0.680
Hemoglobin (mg/dL)	12.8 ± 2.1	13.6 ± 2.8	0.045
Glukoz (mg/dL)	189.1 ± 86.2	91.2 ± 14.3	< 0.001
BUN (mg/dL)	38.9 ± 17.9	35.7 ± 16.6	0.178
Creatinine (mg/dL)	0.99 ± 0.86	1.01 ± 0.75	0.834
Total Cholesterol (mg/dL)	176 ± 44	183 ± 54	0.415
LDL Cholesterol (mg/dL)	103 ± 35	113 ± 44	0.080
HDL Cholesterol (mg/dL)	40.7 ± 13.9	41.9 ± 9.8	0.490
Triglyceride (mg/dL)	173 ± 108	150 ± 101	0.130
HbA1c (%)	8.3 ± 1.9	6.0 ± 0.9	< 0.001
hs-CRP (mg/L)	0.37 ± 0.29	0.42 ± 0.61	0.647
Uric acid (mg/dL)	5.68 ± 1.90	5.55 ± 1.68	0.656

*BUN* Blood urea nitrogen, *DM* Diabetes mellitus, *HDL* high density lipoprotein, *hs-CRP* high sensitive C reactive protein, *LDL* low density lipoprotein

### Vascular ultrasonography findings

A-IMT and C-IMT measurements were successfully performed in all patients who were included in the study. When the B-mode USG findings are examined we found that both IMT values were significantly higher in type II DM patients, with A-IMT being more prominent (Table 2). In DM patients, the frequency of increased A-IMT was significantly and 2 times higher than the control group (Table 2). However, although the increased C-IMT frequency was greater in the DM patient group, this difference was not statistically significant (Table 2).

**Table 2:** Vascular ultrasound finding in patients with type II DM and controls

	Type II DM *n* = 114	Controls *n* = 100	*p*
Common carotid IMT (mm)	0.82 ± 0.18	0.76 ± 0.17	0.023
Abdominal aort IMT (mm)	1.84 ± 0.77	1.49 ± 0.75	0.001
Increased common carotid IMT, n (%)	31 (27.2)	22 (22.0)	0.236
Increased abdominal aort IMT, n (%)	26 (22.8)	11 (11.0)	0.017

*IMT* Intima-media thickness

### Parameters associated with aortic intima-media thickness

Aortic IMT values were found to be strongly and positively correlated with age, systolic blood pressure, blood urea nitrogen, DM diagnosis time and HbA1c levels, and a negatively and significantly correlated with hemoglobin levels (Table 3). Patients with or without oral antidiabetic drug or insulin had similar A- IMT values (*p* > 0.05 both of them). In the linear regression model, the parameters correlating most closely with A-IMT were DM diagnosis time, HbA1c and hemoglobin levels (Table 3).

**Table 3:** The parameters associated with A-IMT and linear regression analysis for parameters significantly correlated with A-IMT

A-IMT	Correlation analyze		Regression analyses	
	*p*	r	*p*	β
C-IMT (mm)	< 0.001	0.896	–	–
Age (year)	< 0.001	0.237	0.081	0.216
Office systolic blood pressure (mmHg)	0.044	0.177	0.201	0.106
DM diagnosis time (years)	< 0.001	0.450	0.001	0.353
Hemoglobin (mg/dL)	0.018	- 0.167	< 0.001	- 0.406
Glukoz (mg/dL)	0.012	0.174	0.756	0.064
HbA1c (%)	0.003	0.202	0.014	0.247
Blood urea nitrogen (mg/dL)	0.003	0.205	0.209	0.141

*DM* Diabetes mellitus, *IMT* Intima-media thickness * ${R}_{Adjusted}^2=0.382$ and C-IMT was excluded in linear regression analyses

### ROC analysis for A-IMT and C-IMT to detect patients with diabetes mellitus

In the ROC analysis, the area under the curve was 0.718 and 0.602 respectively for A-IMT and C-IMT (*p* < 0.05, Table 4). İf A-IMT and C-IMT cut-off values ​​were taken as 1.5 mm and 0.9 mm, respectively, these values detect the patient with DM with a sensitivity of 70.3% and specificity of 63.0%, sensitivity of 60.5% and specificity of 55.8%, respectively (Table 4).

**Table 4:** ROC analysis for A-IMT and C-IMT to detect patients with diabetes mellitus

Variable	AUROC Curve	*p*	Cut-off	Sensitivity	Specificity
A-IMT	0.718 (0.542–0.790)	0.002	1.5 mm	70.3%	63.0%
C-IMT	0.602 (0.506–0.699)	0.012	0.9 mm	60.5%	55.8%

*IMT* Intima-media thickness

## Discussion

The main result of this study is that A-IMT is found to be significantly increased in adult DM patients. Another striking finding is that the A-IMT increase in DM patients is greater than the C-IMT increase. In addition, the parameters most closely associated with A-IMT in our study were found to be DM diagnosis time, serum hemoglobin and HbA1c levels. In addition, as in pediatric DM patients, the A-IMT can easily be measured with new model USG devices in adult DM patients.

Diabetes mellitus causes an increase in smooth muscle cell proliferation in vessel walls, worsens oxidative stress exposure and leads to production of free oxygen radicals [2]. This entire process leads to extensive thickening of the arterial walls. Increased C-IMT is closely associated with asymptomatic atherosclerosis in DM patients and has been proposed as a routine examination [1, 2]. It is a known fact that the B-mode USG evaluation of C-IMT is a reproducible and useful method in the detection of asymptomatic atherosclerosis in DM patients [1, 2]. C-IMT is also associated with CAD, myocardial infarction, and stroke. [11–14]. For these reasons, C-IMT is the preferred method of vascular assessment. C-IMT is also preferred because it is superficially located in neck, easy to visualize, easy to examine, and has many C-IMT documents in the literature.

Aortic IMT is not routinely used in DM patients. The main reasons for this are: (i) There is limited data on the association of A-IMT with macrovascular organ involvement in DM patients [3, 4], (ii) A-IMT assessment is more difficult than C-IMT,(iii) It is not preferred as a routine method in DM disease, (iiii) especially abdominal fat tissue is thought to prevent A-IMT measurement [3]. Atherosclerosis begins to develop as fatty streaks in childhood and can be diagnosed early by sensitive visualization methods [15, 16]. Because atherosclerosis first occurs in the distal abdominal aorta, A-IMT measurement with new USG devices can be used to detect early atherosclerosis development [5–7]. Therefore, we hypothesize that A-IMT obtained with new model high-resolution USG devices may be more useful for early detection of macrovascular complications in adult DM patients than C-IMT. For this reason, we received A-IMT measurements of the abdominal aorta from our patients. Aortic IMT is preferred in pediatric and adolescent groups because tissue penetration is good; target organ damage begins here and is more susceptible to the risk of cardiovascular events. Aortic IMT is thought to be an early indicator of atherosclerosis in this age group. In high-risk children with hypercholesterolemia, DM and inflammatory bowel disease [7, 17–19]. A-IMT is more potent than C-IMT and can detect atherosclerosis earlier. There is no study showing that A-IMT is superior to C-IMT in predicting pre-clinical atherosclerosis, target organ damage, and cardiovascular event risk in adults and most USG devices do not have adequate tissue penetration in adult DM patients. For this reason, A-IMT measurement in adults is not a routine assessment. Diabetes mellitus is the equivalent of CAD and is one of the major risk factors for atherosclerosis. In our study, histopathologic vascular tissue examination was not performed but an explanatory result was obtained for the pathogenesis of macrovascular involvement in DM patients. In our study, the increase of A-IMT in patients with DM is more pronounced and sıgnificant than C-IMT. When performing ROC analyses for A-IMT and C-IMT measurements with DM presence, the limit values for A-IMT and C-IMT with DM presence were 1.5 mm and 0.9 mm respectively. The A-IMT increase was thought to be more prominent than the C-IMT because the atherosclerosis was first detected at the beginning of the distal abdominal aorta, the abdominal aortic vessel diameter and thickness were larger than the carotid artery and C-IMT was affected after A-IMT. According to our evaluation, studies comparing abdominal A-IMT values in adult DM patients are limited in the literature [3, 4]. In a study conducted in adult type 1 DM patients a decade ago, A-IMT evaluations was performed and a lower mean A-IMT value was obtained than our study [3]. However, in this study, it was reported that A-IMT measurement was a difficult challenge and therefore only 69% of the patients had A-IMT measurements. Although there is no clear information on the measurement problem in this study, increased intraabdominal fat tissue, especially in DM patients, is thought to prevent A-IMT measurement. In a recent study of middle-aged sporadic idiopathic hypo parathyroid patients, just like our findings there was a strong correlation between A-IMT and C-IMT [20]. There is a serious difference in the A-IMT measurements obtained in these two studies and there is a reference value problem to standardize the A-IMT. In two small studies including 39 adult DM and 10 congenital adrenal hyperplasia patients, A-IMT measurement was evaluated and the A-IMT value was found to be 0.89 ± 0.17 mm and 1.84 ± 0.68 mm, respectively [3, 21]. Our study was performed with a 5 MHz high-resolution probe and the mean A-IMT value of DM patients was 1.84 ± 0.75 mm. In our study, the A-IMT value obtained in DM patients was very close to Sartorato et al. study [21] but our findings were obtained with more patients than previous study.

There are no detailed clinical studies, and most USG devices do not have probes that sufficiently penetrate tissue in adult DM patients. Hence, A-IMT measurement is not a routine evaluation in adults. With the new USG devices and high-resolution probes, abdominal aorta visualization is much better and A-IMT can be measured by easily. It may be a useful approach to use A-IMT instead of C-IMT to determine subclinical organ damage in type II DM patients.

In our study, we found that both IMT values were significantly higher in type II DM patients, with A-IMT being more prominent. In a previous study, increased C-IMT in type II DM patients was associated with older age, male sex, smoking, and pulse pressure [22]. It has also been reported that advanced glycation end products, which are the result of DM disease, are associated with C-IMT [2]. In our study, we found that the age, systolic blood pressure, increased HbA1c and DM diagnosis time and A-IMT increase were closely related and correlated with the literature. In addition, low hemoglobin levels and increased A-IMT were closely related in our study.

### Limitation

This study investigated the relationship between DM and A-IMT cross-sectionally. We have included a relatively small number of patients, but we have shown that A-IMT is significantly increased in DM patients. DM patients in our study had an average 5 years of diagnosis. This can be very early for the development of macrovascular complications in patients with DM. Because DM is a long-term disease and macrovascular complications occur after many years. For this reason, the follow-up of these patients should continue. The increase in IMT is also related to the cardiovascular events [23]. However, we did not evaluate prognosis in our study. Previous studies have shown that C-IMT is regressed by medical therapy in DM patients. Our study is not a follow-up study and therefore no disease control C-IMT and A-IMT assessments have been performed [24]. There is no study with A-IMT but the C-IMT measurement can be measured automatically and semi-automatically with new software programs, resulting in a lower average value than the manual measurement [25, 26]. This automatic measurement especially removes operator dependence and is more useful for repetitive measurements. However, our high-resolution device did not have this software program so we could not make this evaluation. In our study, if IMT could be measured automatically and semi-automatically, more objective and meaningful results could be obtained. The latest technological devices and high frequency probes are used in our study. For this reason, it may not be possible to obtain similar results with low-frequency and low-resolution devices.

## Conclusions

As in pediatric DM patients, A-IMT is also increased by 22.8% in adult DM patients, and A-IMT can be easily measured with new model USG devices. According to the results of our study, A-IMT, which shows macrovascular organ involvement relatively early in DM, is more useful than C-IMT. A part of the abdominal USG, which is a routine examination in adult DM patients, should include the A-IMT measurement and the A-IMT value should be reported at the end of the abdominal USG. A-IMT is a cheap, easy, reproducible and non-invasive parameter that may be used to detect subclinical atherosclerosis, an early macrovascular complication of adult type II DM.

## Abbreviations

A-IMT: Aortic intima media thickness
BMI: Body mass index
BUN: Blood urea nitrogen
CAD: Coronary artery disease
C-IMT: Carotid intima media thickness
DM: Diabetes mellitus
HDL: High density lipoprotein
hs-CRP: high sensitive C reactive protein
HT: Hypertension
LDL: Low density lipoprotein
USG: Ultrasonography
